# Correlation of Inter-Locus Polyglutamine Toxicity with CAG•CTG Triplet Repeat Expandability and Flanking Genomic DNA GC Content

**DOI:** 10.1371/journal.pone.0028260

**Published:** 2011-12-06

**Authors:** Colm E. Nestor, Darren G. Monckton

**Affiliations:** 1 Institute of Molecular, Cell and Systems Biology, College of Medical, Veterinary and Life Sciences, University of Glasgow, Glasgow, United Kingdom; 2 Breakthrough Breast Cancer Research Unit, University of Edinburgh, Edinburgh, United Kingdom; 3 Institute of Genetics and Molecular Medicine, University of Edinburgh, Edinburgh, United Kingdom; Institut Jacques Monod, France

## Abstract

Dynamic expansions of toxic polyglutamine (polyQ)-encoding CAG repeats in ubiquitously expressed, but otherwise unrelated, genes cause a number of late-onset progressive neurodegenerative disorders, including Huntington disease and the spinocerebellar ataxias. As polyQ toxicity in these disorders increases with repeat length, the intergenerational expansion of unstable CAG repeats leads to anticipation, an earlier age-at-onset in successive generations. Crucially, disease associated alleles are also somatically unstable and continue to expand throughout the lifetime of the individual. Interestingly, the inherited polyQ length mediating a specific age-at-onset of symptoms varies markedly between disorders. It is widely assumed that these inter-locus differences in polyQ toxicity are mediated by protein context effects. Previously, we demonstrated that the tendency of expanded CAG•CTG repeats to undergo further intergenerational expansion (their ‘expandability’) also differs between disorders and these effects are strongly correlated with the GC content of the genomic flanking DNA. Here we show that the inter-locus toxicity of the expanded polyQ tracts of these disorders also correlates with both the expandability of the underlying CAG repeat and the GC content of the genomic DNA flanking sequences. Inter-locus polyQ toxicity does not correlate with properties of the mRNA or protein sequences, with polyQ location within the gene or protein, or steady state transcript levels in the brain. These data suggest that the observed inter-locus differences in polyQ toxicity are not mediated solely by protein context effects, but that genomic context is also important, an effect that may be mediated by modifying the rate at which somatic expansion of the DNA delivers proteins to their cytotoxic state.

## Introduction

A growing number of inherited human diseases have been associated with DNA tandem repeat instability, trinucleotide repeats of the motif CAG•CTG comprising the largest class of such repetitive elements [1]. Expanded CAG•CTG repeat disorders can be further categorised into two principle classes depending upon the location of the array within the associated gene. The first class, which includes myotonic dystrophy type 1 (*dystrophia myotonica* 1, DM1), is defined by a repeat expansion in a non-coding region of the gene. The second class is defined by a polyglutamine (polyQ)-encoding CAG repeat. The unstable CAG polyQ repeat disorders include Huntington disease (HD), spinal and bulbar muscular atrophy, X-linked (SMAX1, also known as SBMA/Kennedy disease), dentatorubral-pallidoluysian atrophy (DRPLA), Machado-Joseph disease (MJD, also known as SCA3), and the spinocerebellar ataxias, 1 (SCA1), 2 (SCA2), 7 (SCA7) and 17 (SCA17). Each of these polyQ disorders is a late-onset neurodegenerative disease associated with the loss of specific neuronal populations [2]. The repeat tracts in the associated gene in all these disorders are typically small (∼5–30 repeats), polymorphic and stably transmitted within the general population. Disease associated alleles in patients have expanded beyond this range and typically contain at least 35 repeats. Although non-coding alleles, such as in the DM1 associated gene, may expand to thousands of repeats, inherited polyQ-coding alleles rarely exceed 100 repeats in humans [2].

Expanded trinucleotide repeat instability is described as a ‘dynamic mutation’, as the frequency and magnitude of length changes vary as the repeat number changes [3]. These dynamic mutations are strongly biased towards expansion in a repeat-length dependent manner, giving rise to increases of allele length from one generation to the next [4], [5], [6]. Moreover, expansions may occur in multiples of repeat units in each step. This contrasts with the instability observed at normal-length polymorphic microsatellites, at which one repeat unit expansions and contractions are equally favoured, resulting in a stable distribution of microsatellite lengths over time [7].

Significantly, at expanded trinucleotide repeats toxicity increases with length, longer repeat tracts resulting in greater levels of cell death and dysfunction in affected tissues, and a more severe phenotype. Thus, intergenerational increases in expanded triplet repeat length are consistent with ‘anticipation’, a clinical characteristic common to these disorders, whereby an earlier age of disease onset and increased severity of symptoms is observed in successive generations [1]. In addition to intergenerational expansion, high levels of age-dependent, expansion-biased, tissue-specific somatic mosaicism are also observed [8], [9]. For example, analysis of post-mortem brain tissue from HD patients has revealed high levels of somatic mosaicism and very large expansions in the striatum, the primary affected tissue in this disorder [10]. Similarly, DM1 patients have significantly longer average allele lengths in muscle compared with blood [11], [12], [13], emphasising the relationship between tissue-specific somatic expansion and pathogenesis. Thus, it has been proposed that whilst intergenerational repeat expansion accounts for the phenomenon of anticipation, somatic expansion is likely to be a major contributing factor in disease progression and the tissue-specificity of symptoms [1].

The precise mechanism(s) underlying the dynamic mutation of CAG•CTG repeats remains unknown. Indeed, transgenic mouse studies have variously implicated a range of DNA repair genes including *Msh2* [14], *Msh3* [15], *Pms2* [16], *Ogg1* [17], *Dnmt1* [18], *DNA ligase 1* [19] and *Xpa* [20]. Surprisingly however, a genome wide analysis did not find a correlation between the steady state DNA repair gene transcript levels and the tissue specificity of repeat instability [21]. Nonetheless, it is clear that two components of the DNA mismatch repair machinery, Msh2 and Msh3, are absolutely required to generate both germ line and somatic mutations in mice [14], [15], [22], [23] suggesting one major pathway, possibly mediated by inappropriate DNA mismatch repair [1]. In addition to obvious *trans*-acting factors involved in governing expanded repeat behaviour such as the mismatch repair system, sex of the transmitting parent and tissue type [1], numerous lines of evidence suggest a major role for *cis*-acting factors in CAG•CTG instability. Expanded CAG•CTG instability is locus-specific, not genome-wide indicating that factors local to the repeat influence its mutability. The most obvious factors are those internal to the array such as number of repeats and sequence purity [24]. However, a growing body of evidence from murine models of CAG•CTG instability also support the involvement of *cis*-elements in the DNA sequences flanking the repeat unit [25], [26], [27], [28], [29], [30]. Likewise, we previously revealed that the intergenerational expandability (a length-normalised measure of propensity toward repeat expansion) of the human disease associated expanded CAG•CTG repeat loci differ significantly from one another, confirming a role for additional *cis*-acting modifiers of repeat stability flanking the repeat [31]. Moreover, we showed that inter-locus variation in expanded CAG•CTG repeat dynamics is strongly correlated with the GC content of the genomic flanking DNA, with the most expandable loci having the highest flanking GC contents [31].

As all the dynamic repeat disorders that possess an expanded polyQ tract are dominant, display a similar inverse relationship between polyQ length and age-at-onset, and lead to progressive neuronal degeneration [2], it appears not unreasonable to suggest that expanded polyQ tracts are inherently toxic and that some aspects of polyQ toxicity may be conserved between disorders. This idea is strengthened by the finding that insertion of a long polyQ encoding tract into the mouse *Hprt* gene can reproduce features of the associated human disorders including a late onset neurological phenotype and neuronal intranuclear inclusions [32]. Similarly, the expression of a long polyQ tract with only 10 flanking amino acids can cause a neurodegenerative phenotype in *Drosophila* [33]. Moreover, protein mis-folding, the formation of polyQ containing aggregates and transcriptional misregulation in affected tissues are molecular abnormalities clearly shared by all the disorders [34].

Despite shared components of the pathogenic pathway, and although all expanded polyQ disorders show a similar inverse relationship between polyQ number and age-at-onset of symptoms, the absolute number of polyQ repeats associated with a given age-at-onset of symptoms varies considerably between the disorders [2]. For example, whereas an age-at-onset of 40 years in MJD typically requires the individual to inherit more than 70 repeats, an inherited allele length of less than 45 repeats will have a similar age-at-onset in SCA2 [2]. These inter-locus differences in polyQ toxicity are widely assumed to be a consequence of the different protein contexts in which each polyQ tract is found in its host protein [34], [35], [36], , resulting in markedly different toxicity thresholds between disorders. Such protein context effects could be mediated by amino acid sequences flanking each polyQ tract modifying the cytotoxic potential of the polyQ tract (*e.g.* by modifying aggregation dynamics), or by effects on the normal function of the protein.

As the size of the native expanded-polyQ containing proteins varies greatly (41 kDa–347 kDa), their primary sequences are not similar, and the position of the tract relative to the translation start site differs, the polyQ tracts clearly do have very different protein contexts. Indeed, there is ample evidence that changing the flanking amino acid sequence of an expanded polyQ tract can change its pathogenic potential. For instance, studies in yeast showed that altering the flanking sequence of an expanded HD *HTT* exon 1 fragment, by the simple addition of a FLAG-tag, caused a previously non-toxic fragment of HTT exon 1 to induce characteristic length-dependent polyQ toxicity [39]. Similarly, deletion or replacement of the Josephin domain of expanded polyQ-containing ATXN3 significantly reduced the propensity of the protein to form aggregates [40], as did deletion or replacement of the AXH domain of the ATXN1 protein [35]. Other findings suggest that polyQ protein context could mediate cytotoxicity by affecting the ability of the ubiquitin-proteasome system to target and clear the cell of toxic expanded proteins and aggregates [41], [42]. Similarly, polyQ toxicity can be modified by the phosphorylation status of flanking amino acids. Strikingly, replacement of a single serine phosphorylation site with an alanine residue in an ATXN1 transgene with a long polyQ tract dramatically reduces toxicity [43], while conversely replacement of the same serine with a phospho-mimetic aspartic acid residue renders a wild type ATXN1 transgene with a short polyQ tract cytotoxic [44]. Indeed, there is mounting evidence that some of the pathological effects of polyQ expansions can be mediated through a gain of normal protein activity [45]. Thus, protein context can have a major effect on polyQ toxicity and it seems very likely that some of the observed difference in inter-locus polyQ toxicity will be attributable to protein context effects. However, other than to say that certain protein contexts are more or less toxic, taken together the known protein context effects offer no quantifiable rationalisation of the observed inter-locus differences in polyQ toxicity.

It is our primary hypothesis that ongoing somatic expansion contributes toward disease progression in the repeat expansion disorders [1]. Under such a scenario the age-at-onset of symptoms in an individual can be rationalised as the product of a sufficiently high proportion of cells having acquired a sufficiently large repeat tract to mediate tissue dysfunction. Therefore, any major modifier of the dynamics of somatic expansion should also modify the age-at-onset of symptoms. Consequently, for a group of disorders, such as the polyQ disorders, that share some aspects of a common downstream pathogenic pathway, differences in the underlying somatic stability of the expanded repeat should result in differences in the relationship between the number of repeats inherited and age-at-onset. We thus hypothesised that the rate at which somatic expandability delivers polyQ proteins to their cytotoxic state would be a critical factor in expanded polyQ-disease pathogenesis, and might contribute toward the observed inter-locus differences in polyQ toxicity. Here we tested this hypothesis, by quantifying the relationship between inter-locus polyQ toxicity, CAG•CTG repeat expandability and flanking DNA GC content.

## Results

### Defining inter-locus polyQ toxicity

**Figure 1:**
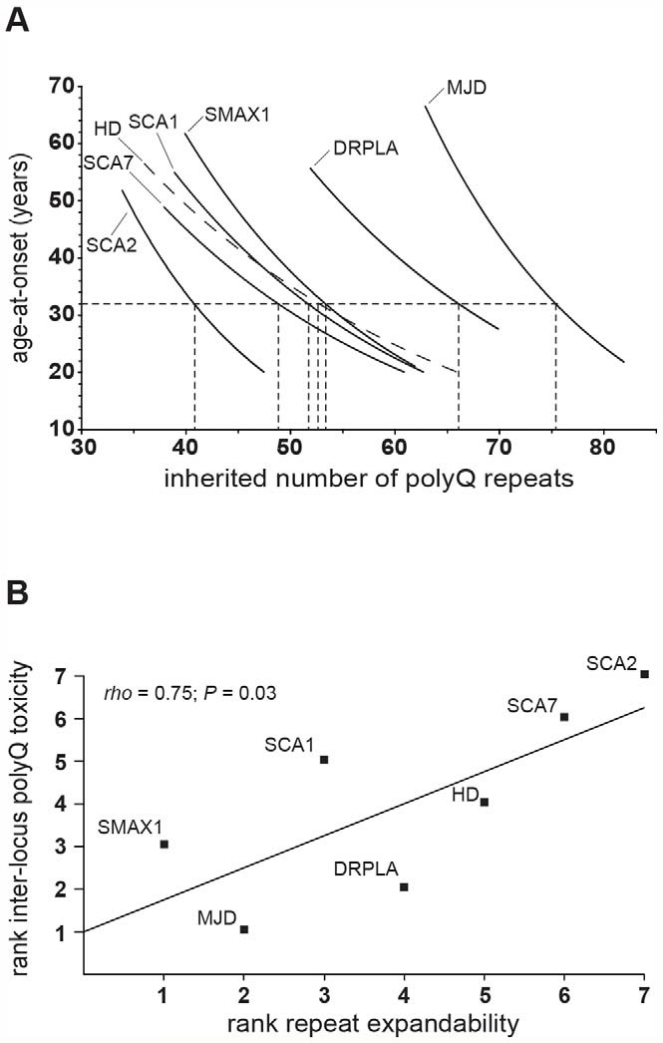
Repeat expandability correlates with inter-locus polyQ toxicity. (**A**) The graph shows the exponential decay regression lines fitted to the age-at-onset and inherited repeat length distributions in the polyQ disorders (Huntington disease (HD)(dashed line), spinal and bulbar muscular atrophy, X-linked (SMAX1), dentatorubral-pallidoluysian atrophy (DRPLA), Machado-Joseph disease (MJD), spinocerebellar ataxia 1 (SCA1), 2 (SCA2) and 7 (SCA7)). The inter-locus polyQ toxicities were derived from the parameters of the regression line of each disorder for the modal age-at-onset of 32 years (dashed lines). (**B**) Plot of ranked expandability and ranked inter-locus polyQ toxicity at the modal age-at-onset (32 years) with the regression line (one-tailed Spearman's rank; *rho* = 0.75; *P* = 0.03; N = 7).

In order to investigate the factors mediating differences in polyQ toxicity, we sought to develop a robust quantitative measure of inter-locus polyQ toxicity. Previously, Gusella and MacDonald analysed published studies to collate measured repeat length versus age-at-onset data from large numbers of patients with the polyQ disorders [2]. The data set comprises measured polyQ length and age-at-onset for over 2,400 individuals with at least 100 patients for each disorder. Although patients may acquire very large somatic expansions in the affected brain region [10], levels of somatic mosaicism in the peripheral tissues used for genotyping are generally very low [8], [46], [47] such that we can assume that the measured allele length represents the inherited repeat length. Using these data, we carried out a detailed statistical analysis of the nature of the relationship between inherited repeat number and age-at-onset both within and between the seven dynamic DNA polyQ disorders. Firstly, we determined that the majority of individuals with these disorders initially develop symptoms in adult life, with a modal age-at-onset of 32 years. Juvenile cases, with an age at onset under 20 years, are relatively rare, but develop an extreme phenotype that is very similar between the disorders and in which the well defined regional specificity of the adult onset neuropathology is lost [48], [49], [50], [51]. Moreover, because of this extreme differential phenotype and the paucity of juvenile onset data for most of these disorders, cases with an age-at-onset under 20 years of age were excluded from the analyses. Testing a range of curve estimation regression models, an exponential decay function was found to best describe the relationship between age-at-onset and repeat number for all disorders. Subsequently, we used the parameters derived from the regression analysis for each disorder to calculate the inherited repeat number predicted to result in an age-at-onset of 32 years, the modal age-at-onset (Figure 1A, Table 1, and Figure S1). We propose that the repeat numbers thus obtained, represent a robust quantitative measure of the relative inter-locus polyQ toxicity confirming SCA2 and SCA7 as the most toxic, and DRPLA and MJD as the least toxic, polyQ expansions. Although some earlier, small-scale studies reported a simple linear relationship between age-at-onset and repeat length it is widely recognised that the relationship is best modelled by an exponential function [2], [52], [53], [54]. In any case, modelling the relationship between age-at-onset and repeat length using a simple linear function generated similar values of relative inter-locus toxicity (Figure S2A).

**Table 1:** Inter-locus polyQ toxicity and expandability of the dynamic DNA polyQ loci.

disorder	*gene*	*r* a	inter-locus polyQ toxicity^b^ (95% C.I.)	rank toxicity	expandability^c^	rank expandability
MJD	*ATXN3*	0.52	75.4 (75.2–76.4)	1	0.05	2
DRPLA	*ATN1*	0.21	66.1 (64.8 - NC)	2	0.11	4
SMAX1	*AR*	0.39	53.3 (52.3–57.1)	3	0.03	1
HD	*HTT*	0.40	52.2 (51.2–52.7)	4	0.14	5
SCA1	*ATXN1*	0.63	51.7 (51.2–52.5)	5	0.08	3
SCA7	*ATXN7*	0.39	48.8 (47.8–50.0)	6	0.72	6
SCA2	*ATXN2*	0.41	40.8 (40.5–41.9)	7	0.83	7

a the coefficient of correlation (*r*) of age-at-onset versus repeat length was obtained by fitting an exponential decay model to each dataset (see Figure 1 & Figure S1). All correlations were highly significant (*P*<0.001).

b repeat length corresponding to an age at onset of 32 years.

c sex-averaged intergenerational expandability of each disorder as previously described [31].

CI; Confidence Interval.

### Inter-locus polyQ toxicity correlates with repeat expandability

We hypothesised that the rate at which somatic expandability delivers polyQ proteins to their cytotoxic state would be a critical factor in expanded polyQ-disease pathogenesis, and contributes towards the observed inter-locus differences in polyQ toxicity. Thus, we predicted that inter-locus polyQ-toxicity would be positively correlated with locus expandability; the more toxic polyQ loci would be those with the highest expandability. Taking into account the effect of progenitor allele length, we previously quantified observed differences of intergenerational variability between expanded CAG•CTG repeat loci; calculating the relative expandability of each locus using pedigree data gleaned from the literature (Table 1) [31]. Employing these values of sex-averaged expandability we found that inter-locus polyQ toxicity and locus expandability were significantly correlated using a rank order test (one-tailed Spearman's rank; *rho* = 0.75; *P* = 0.03; N = 7) (Figure 1B). Similarly significant correlations were obtained when an age-at-onset of 30 (one-tailed Spearman's rank; *rho* = 0.75; *P* = 0.03; N = 7), 40 (one-tailed Spearman's rank; *rho* = 0.82; *P* = 0.02; N = 7) or 50 (one-tailed Spearman's rank; *rho* = 0.82; *P* = 0.02; N = 7) years was used to determine inter-locus polyQ toxicity, suggesting that the inter-locus polyQ toxicity values as determined at 32 years age-at-onset are broadly representative of the relationship between the variables throughout the dataset as a whole. Again, a similarly significant relationship between inter-locus polyQ toxicity and locus expandability (one-tailed Spearman's rank; *rho* = 0.75; *P* = 0.03; N = 7) was obtained when using inter-locus toxicity values derived from a linear model of the relationship between repeat length and age-at-onset (Figure S2B).

**Figure 2:**
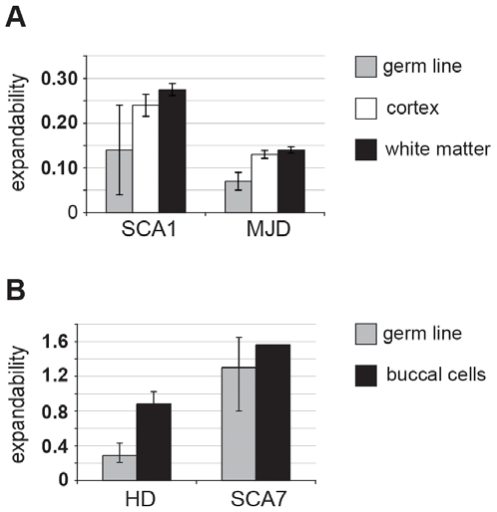
Intergenerational instability is predictive of somatic instability. (**A**) Repeat-length normalised levels of somatic mosaicism in the brains of SCA1 and MJD patients is similar to the levels of germ line instability observed in these disorders. Data were obtained from meta-analysis of a published study of somatic mosaicism in the cerebral cortex (N_MJD_ = 11, N_SCA1_ = 7) and white matter (N_MJD_ = 9, N_SCA1_ = 6) of SCA1 and MJD individuals (Table S1) (Maciel *et al*, 1997). (**B**) Repeat-length normalised levels of somatic mosaicism in buccal cells of HD and SCA7 patients is similar to the levels of germ line instability observed in these disorders. Data were obtained from meta-analysis of published studies of somatic mosaicism in the buccal cells of HD (N = 12) [8] and SCA7 (N = 1) [34] individuals (Table S2).

As detailed quantitative data on somatic instability are not available for most of the polyQ disease loci, we have used our previously derived measure of the relative expandability of the repeat tract based on intergenerational transmissions [31]. Nonetheless, expanded CAG•CTG repeat transgenic mouse models have revealed that mouse lines showing the greatest intergenerational expandability also exhibit higher levels of somatic expandability (*e.g.* [25], [26], [28], [29]) suggesting that the two measures are comparable. The limited human data that are available also support a similar relationship. Examining data from a published study of somatic expandability in post-mortem brain tissue of SCA1 and MJD patients [55], we found that the repeat-length normalised levels of somatic expandability in SCA1 were approximately double the levels found in MJD in both cerebral cortex (N_MJD_ = 11; N_SCA1_ = 7; Mann-Whitney U = 0; *P*<0.0001) and cerebral white matter (N_MJD_ = 9; N_SCA1_ = 6; Mann-Whitney U = 0; *P*<0.001); similar to the relative levels of germ line expandability observed in these disorders (Figure 2A and Table S1) [31]. Similarly, meta-analysis of published studies of somatic expandability in buccal cells of HD [8] and SCA7 [47] patients, revealed that the repeat-length normalised levels of somatic expandability in SCA7 were significantly greater than those in HD (N_HD_ = 12; N_SCA7_ = 1; T-test = −9.58; *P*<0.0001). Again, the levels of somatic expandability in HD and SCA7 were comparable to the levels of germ line expandability observed in these disorders (Figure 2B and Table S2) [31], suggesting that relative intergenerational expandability is an accurate proxy of relative somatic expandability.

### Refining the association between CTG•CAG expandability and flanking genomic DNA GC content

**Figure 3:**
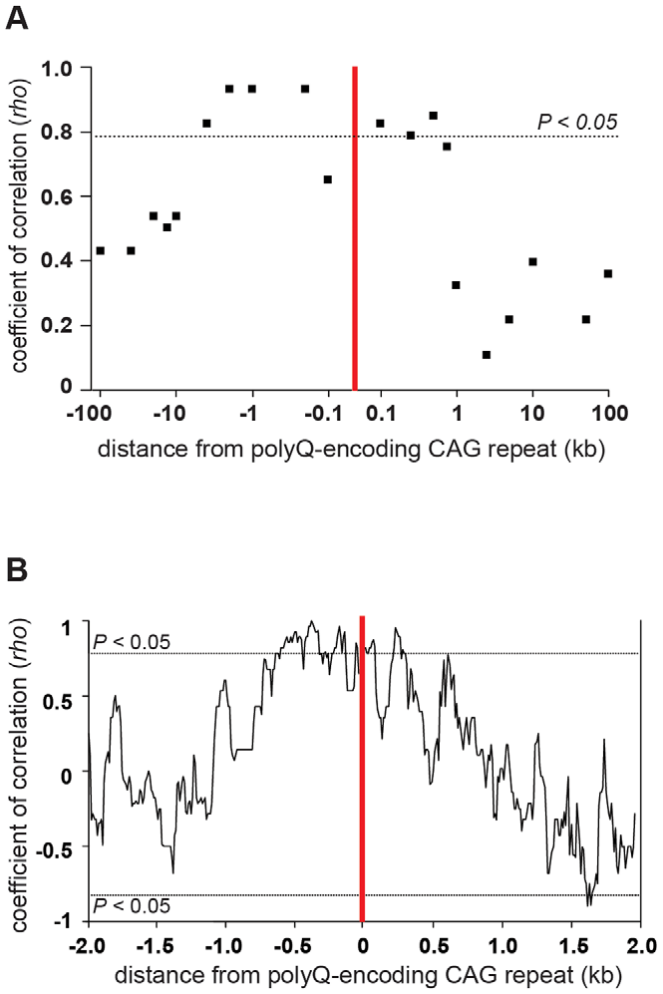
Repeat expandability correlates with flanking genomic DNA sequence GC content. (**A**) polyQ-encoding CAG-repeat expandability correlates with proximal, but not distal flanking genomic DNA sequence GC content. Distance from the repeat (red vertical line) is plotted on a log scale against Spearman's coefficient of correlation (*rho*) with expandability [31]. The dashed line shows the threshold for statistical significance (*P*<0.05; two-tailed). (**B**) The graph shows the coefficient of correlation of flanking genomic DNA GC content of the seven dynamic DNA CAG polyQ-encoding loci with repeat expandability. Spearman's rank coefficient of correlation (*rho*) was calculated to a distance of 2,000 bp both 5′ and 3′ of each repeat using a sliding window of 100 bp and step size of 10 bp. The dashed line shows the threshold for statistical significance (*P*<0.05; two-tailed). and The position of the CAG•CTG repeat is represented by the vertical red bar.

We previously described a significant positive correlation between repeat expandability and the GC content of genomic DNA flanking sequences and postulated that flanking GC content directly modifies repeat stability [31]. When we first conducted this analysis the human genome sequencing project was unfinished and many flanking sequences were absent or incomplete. Here, employing the latest assembly of the human genome (NCBI 36), we characterised this relationship in finer detail and to a greater distance from each locus. Employing the seven polyQ loci a significant rank correlation between sex-averaged germ line expandability and flanking genomic DNA GC content was found up to a distance of 1,000 bp from the repeat when the combined flanking sequences of the loci were analysed (Table 2). Statistically significant correlations were also obtained when the 5′ and 3′ flanking sequences were analysed independently. The absence of any significant association at distances from 1 kb to 100 kb suggests that the observed correlations proximal to the repeats are not a simple function of the wider chromosomal GC content surrounding each locus (Figure 3A).

**Table 2:** Correlation of flanking genomic DNA GC content with repeat expandability of the polyQ loci.

	5′ flankingsequence		3′ flankingsequence		combined flanking sequence	
distance from repeat	*rho* a	*P* b	*rho*	*P*	*rho*	*P*
100,000 bp	0.429	0.337	0.357	0.432	0.357	0.337
50,000 bp	0.429	0.337	0.214	0.645	0.214	0.645
10,000 bp	0.536	0.215	0.393	0.383	0.321	0.482
5,000 bp	0.500	0.253	0.214	0.645	0.214	0.645
2,500 bp	0.536	0.215	0.107	0.819	0.357	0.432
1,000 bp	0.821	0.023*	0.321	0.482	0.929	0.003*
750 bp	0.929	0.003*	0.750	0.052*	0.929	0.003*
500 bp	0.929	0.003*	0.847	0.016*	0.929	0.003*
250 bp	0.929	0.003*	0.786	0.036*	0.786	0.036*
100 bp	0.649	0.115	0.821	0.023*	0.786	0.036*

a Spearman's rank coefficient of correlation.

b *P*-value of Spearman's rank coefficient of correlation.

*statistically significant at *P*<0.05.

In order to further describe the area of significant association flanking the loci, we determined a continuous GC content profile of the genomic DNA flanking the polyQ loci to a distance of 2 kb from the repeat using a sliding window of 100 bp and step size of 10 bp. Subsequently, the rank correlation of GC content with the expandability of all loci was determined along the flanking sequences at each 10 bp interval. These data confirmed that the region of significant correlation was restricted to <±1 kb. Interestingly, a substantial difference in the correlation profile of the 5′ and 3′ sequences immediately adjacent to the loci was evident. The 5′ sequence shows an almost continuous significant correlation (N = 7; *P*<0.05) from a distance of 140 bp to 850 bp from the loci, whereas a more punctuated profile was found 3′ of the repeat array (Figure 3B).

### Inter-locus polyQ toxicity correlates with flanking genomic DNA GC content

**Figure 4:**
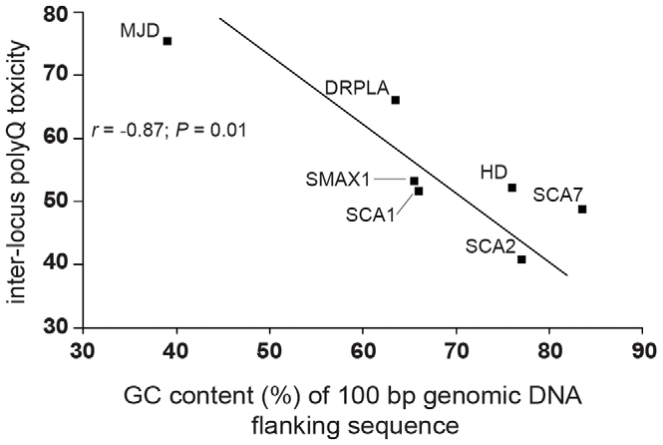
Inter-locus polyQ toxicity correlates with genomic DNA flanking sequence GC content. The graph shows the regression analysis between inter-locus polyQ toxicity and the GC content of the genomic DNA flanking sequences at a distance of 100 bp (*r* = −0.87; *P* = 0.01; N = 7).

**Figure 5:**
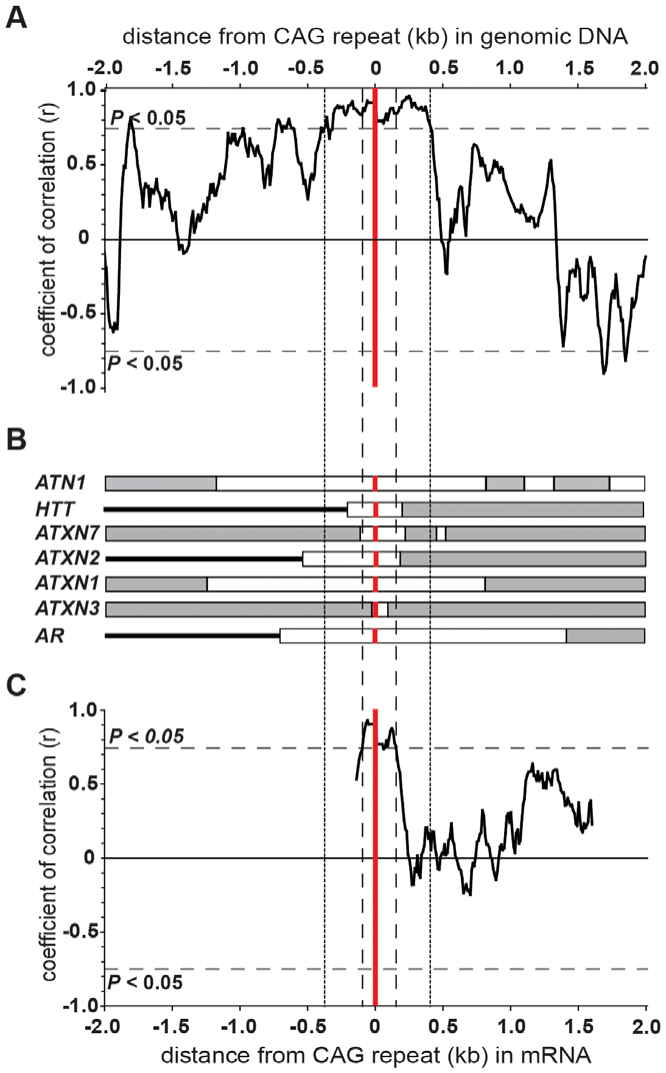
Inter-locus polyQ toxicity correlates with the flanking genomic DNA sequence GC content, but does not extend beyond the repeat containing exon in the mRNA sequence. (**A**) Inter-locus polyQ toxicity correlates with the flanking genomic DNA sequence GC content. The graph shows the coefficient of correlation (*r*) for the relationship between inter-locus polyQ loci toxicity and flanking genomic DNA sequence GC content. GC content was sampled using a sliding window of 100 bp and a step size of 10 bp. The threshold for statistical significance (dashed lines) and the position of the CAG•CTG repeat (red vertical bar) are also shown. Note that the region of statistically significant correlation extends for ∼400 bp either side of the repeat tract (as indicated by the vertical dotted lines). (**B**) Gene structure of the seven polyQ containing genes. All diagrams are to scale. Exons (white box), introns (grey box), intergenic regions (horizontal black bar), and repeat tract (vertical black bar) are shown. (**C**) Inter-locus polyQ toxicity only correlates with flanking mRNA sequence GC content to the 5′ and 3′ ends of their host exons. The graph shows the coefficient of correlation (*r*) for the relationship between inter-locus polyQ toxicity and flanking mRNA sequence GC content determined as in (A). Note that the region of statistically significant correlation extends for only ∼100 bp either side of the repeat tract (as indicated by the vertical dashed lines) corresponding to the length of mRNA sequence encoded by the repeat containing exons and not extending into flanking exons.

If repeat stability is indeed a major modifier of inter-locus polyQ toxicity, and flanking genomic DNA GC content governs repeat stability, a strong association between inter-locus polyQ toxicity and flanking genomic DNA GC content would be expected. Applying the same methodology, we analysed the association of flanking genomic DNA GC content with inter-locus polyQ toxicity. As we possess reliable quantitative data for both GC content and inter-locus polyQ toxicity a product-moment correlation (Pearson, *r*) was performed. A statistically significant correlation between inter-locus polyQ toxicity and flanking genomic DNA GC content was observed from 100 bp (Pearson's *r* = −0.87, *P* = 0.015) (Figure 4) to approximately 400 bp flanking the repeat tract (Figure 5A). A similar highly significant association with flanking genomic DNA GC content was observed both 5′ and 3′ of the CAG repeat loci (Figure 5A).

### Inter-locus polyQ toxicity does not correlate with the GC content of the mRNA or location within the gene

We considered it possible that the observed CAG repeat genomic DNA flanking sequence GC content correlation with inter-locus polyQ toxicity could reflect effects mediated at the level of the mRNA. Employing manually curated RefSeq mRNA sequences for each gene, we investigated the association between inter-locus polyQ toxicity and mRNA GC content. No significant correlation between inter-locus polyQ toxicity and total mRNA GC content was found (*r* = −0.28; *P* = 0.58; N = 7). Employing the sliding window approach as before, a significant correlation between flanking mRNA sequence and GC content was only found immediately proximal (<100 bp) to the repeat tract and dropped rapidly thereafter (Figure 5C). This small region of significant correlation corresponds closely to the region of sequence defined by the 5′ and 3′ boundaries of the repeat-containing exons in each gene and does not extend into the sequence coded for by adjacent exons (Figure 5B) suggesting that the correlation between flanking genomic DNA GC content and inter-locus polyQ toxicity does not reflect effects mediated at the level of the mRNA. Additionally, inter-locus polyQ toxicity did not correlate significantly with the distance of the repeat tract from either the transcription start site (Spearman's rank correlation; N = 7, *rho* = 0.43, *P* = 0.3) or translation start site (Spearman's rank correlation; N = 7, *rho* = 0.5, *P* = 0.22).

### Inter-locus polyQ toxicity does not correlate with flanking protein sequence properties

Although no correlation was observed with the GC content of the mRNA, we considered it possible that the observed correlation between CAG repeat DNA flanking GC content and inter-locus polyQ toxicity could reflect the GC content of codons encoding amino acids with polyQ toxicity mediating properties. Protein properties were quantified using published, experimentally and empirically derived scales of protein physiochemical characteristics (Table S3). Employing these scales of predicted amino acid composition, flexibility, hydrophobicity, and polarity, no correlation with inter-locus polyQ toxicity was identified (Figure S3). Similarly, no correlation between predicted secondary structural features flanking the polyQ tract (alpha helices, beta sheets, beta turns or coils) and inter-locus polyQ toxicity was found (Figure S4). Interestingly, several secondary structure prediction algorithms [36], [56], [57], [58] failed to identify any regions of conserved structure in the sequences flanking the polyQ repeat in each protein (data not shown) suggesting polyQ toxicity is not dependent on a particular local structural context. Finally, inter-locus toxicity does not correlate with the GC content of the 1st and 2nd codon positions (which will correlate well with amino acid identity) flanking the repeat, but does correlate with the 3rd codon GC content (which will not correlate well with amino acid identity), but only for the region encompassed by the immediate flanking exon (Figure S5).

### Inter-locus polyQ toxicity does not correlate with transcript levels

**Figure 6:**
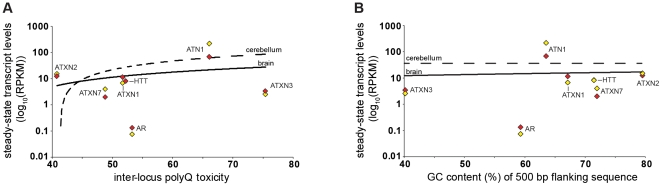
Steady-state transcript levels in human brain do not correlate with inter-locus toxicity or flanking DNA GC content. (**A**) Correlation (Pearson, *r*) between inter-locus toxicity and polyQ gene steady-state transcript levels in whole brain (*r* = 0.33, *P* = 0.47; yellow diamond) or cerebellum (*r* = 0.37, *P* = 0.31; red diamond). (**B**) Correlation (Pearson, *r*) between 500 bp flanking DNA GC (%) content and polyQ gene steady-state transcript levels in whole brain (*r* = 0.07, *P* = 0.89) or cerebellum (*r* = 0.34, *P* = 0.46). Similarly, no significant correlation was observed between polyQ gene steady-state transcript levels and 100 bp flanking DNA GC (%) content (brain, *r* = −0.07, *P* = 0.89; cerebellum, *r* = −0.09, *P* = 0.85) or 2000 bp flanking DNA GC (%) content (brain, *r* = 0.37, *P* = 0.41; cerebellum, *r* = 0.34, *P* = 0.46). Steady-state transcript levels values are averages of values from multiple independent samples of normal human whole brain (*N* = 2, yellow diamond) and cerebellum (*N* = 6, red diamond). The least squares linear regression lines are shown for whole brain (solid) and cerebellum (dashed). Steady-state transcript levels were calculated as ‘reads per kilobase of exon model per million mapped reads’ (RPKM) [59]. RPKM values are shown in log_10_ scale for.

The polyQ expansions are located in various positions within each associated gene, often very distant from the promoter. In addition, the correlation with GC content extends only a short distance from the repeat. Nonetheless, given the known association between GC content and expression levels, it is possible that the correlation with GC content reflects an effect mediated by gene expression levels. Thus, we tested if steady state transcript levels correlated with either the polyQ toxicity or the GC content flanking the repeat. To investigate the relationship between inter-locus polyQ toxicity and inter-locus polyQ gene expression levels, we analysed recently published RNA-seq (next generation sequencing of RNA) data of human brain [59]. We found no significant correlation between inter-locus polyQ toxicity and polyQ gene expression in either normal human whole brain (*r* = 0.33, *P* = 0.47, *N* = 7) or normal human cerebellum (*r* = 0.37, *P* = 0.41, *N* = 7), (Figure 6A). Similarly, no significant correlation between flanking sequence GC content and expression was observed (Figure 6B).

## Discussion

Although the inverse relationship between age-at-onset and repeat length is broadly similar in the dynamic DNA polyQ disorders, the inherited number of repeats required to cause a given age-at-onset varies dramatically between disorders (Figure 1) [2]. Notably, the SCA2 polyQ expansion is almost twice as toxic as the MJD expansion and there is virtually no overlap in the repeat length distributions observed in the patient populations. Understanding the basis of these dramatic contextual differences could be important in the development of therapies. For instance, if the effect of the protective environment of the MJD repeat could be replicated in SCA2, then individuals with SCA2 alleles less than 60 repeats (>99% of patients), might never develop symptoms. It has been widely assumed that this inter-locus polyQ toxicity difference is due to protein context mediated effects on polyQ tract cytotoxicity [34], [35], [36], [37], [38]. Indeed, numerous studies have shown that protein context can be a major modifier of polyQ tract toxicity (*e.g.* [35], [39], [40], [41], [42], [43], [44], [45]). However, until now, no rationalisation of how protein context determines the considerable observed inter-locus polyQ toxicity has been described. We hypothesised that the rate at which somatic expansion delivers a polyQ protein to its cytotoxic state would be a critical factor in expanded polyQ-disease pathogenesis, and could also contribute toward the observed inter-locus differences in polyQ toxicity. Employing age-at-onset data for seven of the polyQ disorders we quantified the inter-locus polyQ toxicity differences and found that the inter-locus polyQ toxicity is indeed significantly correlated with the underlying expandability of the CAG repeat tract. Moreover, we showed that the flanking GC content also correlates highly with inter-locus polyQ toxicity. However, as GC content and repeat expandability are correlated, it is possible that expandability and polyQ toxicity are both dependent variables of GC content and are not linked in a direct cause and effect pathway. Thus, we sought to explore the alternative explanations for this observation. Importantly, we found that inter-locus polyQ toxicity is not correlated with the GC content of the mRNA, the properties of the amino acid sequence, or with the position of the repeat tract within the gene or protein. These data appear to preclude the theory that the observed correlation between GC content and inter-locus polyQ toxicity is mediated by the immediate flanking amino acid sequence of the polyQ tract. Of course, these results do not preclude a role for broader protein context effects in mediating inter-locus polyQ toxicity, but suggest that such effects are more likely to be mediated by larger more complex protein domains whose signature is not reflected in the flanking sequence of the genomic DNA. This interpretation is consistent with the mounting evidence indicating an important role for gain of wild-type function in the polyQ disorders [45]. Given the known association between GC content and promoter activity, we also considered it possible that the correlation between GC content and inter-locus polyQ toxicity might be mediated by gene expression levels: high expression levels of a smaller polyQ expansion might be as toxic as lower expression of a larger expansion. To test this hypothesis, we used recently derived RNA deep sequencing data to test the correlation between inter-locus polyQ toxicity and the relative steady state transcript levels in human brain and cerebellum. These analyses showed that neither polyQ toxicity or the GC content of the repeat flanking DNA were correlated with steady state transcript levels. Again, these data do not preclude a role for expression levels in mediating some aspects of inter-locus polyQ toxicity, but indicate that transcription levels are not the basis of the observed correlation of GC content with inter-locus polyQ toxicity. Thus, the most logical explanation of the data is that the significant correlation between flanking DNA GC content and inter-locus polyQ toxicity is a consequence of flanking GC content effects on DNA repeat stability *i.e.* that the age-at-onset of an individual with a polyQ disorder is a function of the inherited allele length coupled with the rate at which it expands somatically, and that the somatic expansion rate is mediated by the GC content of the flanking DNA.

We previously detailed a significant association between flanking GC content and locus expandability [31]. Here, analyzing this relationship in finer detail and at greater distances from each locus, we found a significant positive correlation between proximal flanking GC content and repeat instability for the polyQ-encoding CAG. The data presented here confirm that the effect is local, limited to within ±1 kb of the repeat tract, excluding an effect mediated by high order isochore domains. Flanking GC content may affect repeat stability by modifying the formation or stability of the presumptive slipped strand DNA intermediates [60] or the DNA-RNA hybrid R-loops that have been implicated in generating them [61]. Alternatively, the GC content of the flanking DNA may modify the downstream processing of aberrant structures by the DNA repair machinery either directly through its effect on the biophysical properties of DNA or through CpG methylation effects on transcription and/or chromatin dynamics such as CTCF-binding [30]. The effect of GC content on repeat expandability could be directly tested in mutant mice in which the flanking DNA sequence GC content is altered, without altering the amino acid sequence in the mature protein.

Mutant polyQ-encoding CAG tracts also cause the atypical disorders SCA6 and SCA17. However, neither can be classified as a dynamic mutation since both loci are genetically relatively stable. Even ‘expanded’ SCA6 alleles are relatively small (typically 20–30 repeats), and there is some debate as to whether SCA6 represents a true polyQ repeat disorder. Although the carboxy terminus can form polyglutamine aggregates [62], SCA6 has a distinct neurochemical profile from SCA1 and SCA2 [63] and truncating mutations in the same SCA6 associated *CACNA1A* calcium channel gene cause the highly overlapping episodic ataxia type 2A phenotype [64]. Although expanded CAG repeat SCA17 alleles are relatively long (typically 50–60 repeats), they are nearly always interrupted by stabilising CAA codons [65]. Nonetheless, very rare cases of unstable pure SCA17 repeat tracts have been reported [66], [67], [68], [69]. Although the data for pure SCA17 repeat transmissions are too limited (*N* = 9) for inclusion in the main analyses presented in this study, we decided to test whether the relationship between expandability, inter-locus polyQ toxicity and flanking DNA GC content of unstable SCA17 alleles fitted with our model of somatic mosaicism mediated inter-locus polyQ toxicity. SCA17 data was obtained by meta-analysis of published cases of unstable SCA17 alleles (Table S4). Interestingly, inclusion of data from unstable SCA17 loci resulted in a more significant correlation between expandability and inter-locus polyQ toxicity (one-tailed Spearman's *rho* = 0.74; *N* = 8; *P* = 0.02) (Figure S6A and S6B). Moreover, the highly significant association between inter-locus polyQ toxicity and flanking genomic DNA GC content is maintained upon inclusion of the SCA17 locus (Figure S6C).

The data we have presented here further support a role for somatic expansion in the dynamic DNA disorders. The hypothesis that somatic expansion of repeats contributes towards age-at-onset of symptoms and disease progression is further supported by a number of observations in patients. For instance, individuals with expanded yet stable SCA1 alleles exhibit significantly delayed onset of symptoms [70], [71] or remain asymptomatic [72]. These individuals contain histidine-encoding CAT interruptions in the expanded CAG repeat. SCA1 alleles containing interruptions tend not to expand, whereas loss of repeat interruptions is associated with repeat expansion [73]. However, it should also be noted that the presence of histidines with the polyQ tract can also alter aggregation dynamics [74]. Similarly, CAA interrupted CAG expansions in *ATXN2* do not induce SCA2, but instead produce a Parkinsonian phenotype, despite the fact that both the pure and interrupted repeat tracts encode pure polyQ [75]. Likewise, a large group of HD patients from Crete with expanded, but for as yet unknown reasons stable HD alleles, had a median age-at-onset 15–20 years later than expected [76]. Significantly, the CAG repeat tract in these patients is also uninterrupted, coding for a pure polyQ tract [77], further implicating repeat instability, not polyQ toxicity, as the major modifier of disease progression. Most convincingly, a recent study of somatic instability in the cortex of HD individuals with expanded repeat tracts of similar length found that somatic instability was a significant predictor of age at onset [78]. The dependence of disease onset and progression on CAG repeat expandability could be readily tested in transgenic mice carrying either a somatically unstable pure CAG repeat tract or a stable CAA/CAG repeat tract, both of which code for a pure polyQ tract in the mature protein. It has already been demonstrated that a genetically stable mixed CAA/CAG transgene can illicit an HD like phenotype, suggesting that somatic expansion is not essential to mediate pathology [79]. However, it needs to be considered that the multicopy transgene used in this study was ∼three fold overexpressed and contained 97 glutamine repeats, more than twice the size of the typical adult onset HD allele (∼45 repeats [2]). Balancing the size of the repeat inserted with the limited lifespan of the mouse and the consequent relatively limited window for somatic expansion may prove problematic. It may thus be necessary to generate a matched allelic series of knock-in mice with different repeat lengths to investigate the relative importance of somatic instability.

Our model of expansion-mediated disease pathogenesis is further supported by a recent computational study which predicted that repeat expansion in somatic tissue determines both age-at-onset and the rate of disease progression [53]. Employing mathematical modeling and computer simulations, it was shown that the more rapid disease progression observed in juvenile cases and the similar age-at-onset, but more rapid disease progression observed in individuals homozygous for polyQ expansions could be accurately represented by a somatic-expansion model, but not by a cumulative polyQ toxicity model [53]. This mathematical model would directly predict that the age of onset relationships for each disease would be shifted as we have revealed by locus-specific effects on mutational dynamics.

These data further support the concept that somatic expansion makes a substantial contribution to disease progression. As such, treatments that resulted in a suppression of repeat expansion would be expected to be therapeutically beneficial [1]. Given the critical role played by Msh2 and Msh3 in the expansion pathway [14], [15], these proteins present themselves as potential therapeutic targets. Indeed, the introduction of an *Msh2* null allele in a knock-in HD mouse model resulted in a 5-month delay in the appearance of aggregates [80]. Although suppression of *Msh2* would be expected to lead to a cancer predisposition phenotype [81], *Msh3* knockouts are not cancer prone [82].

We have revealed a significant association between inter-locus polyQ toxicity and both repeat expandability and the GC content of the flanking DNA. These data provide the first quantitative insights into how to rationalise the observed dramatic differences in inter-locus polyQ toxicity. Of course, these data do not preclude a role for protein context in also contributing toward inter-locus polyQ toxicity and, given the dramatic effects on polyQ toxicity observed *in vitro*, it would be a major surprise if they did not. Likewise, gene expression levels and the tissue-specificity of gene expression patterns would also be expected to contribute. Nonetheless, the coefficient of correlation between GC content and inter-locus polyQ toxicity, r = −0.76, suggests that flanking GC content accounts for ∼57% of the considerable inter-locus variation in polyQ toxicity. Assuming that a sizeable proportion of this effect is mediated via effects on repeat stability, then these data provide the first quantitative insights into how effective therapies that stabilised the repeat tract might be.

## Methods

All genomic DNA analyses used the NCBI 36 (November 2005) assembly of the human genome, obtained from the Ensembl web server (url: http://www.ensembl.org/index.html). The accession numbers of the mRNA sequences employed for each disorder were; NM_001007026 (*ATN1*), NM_000332 (*ATXN1*), NM_002973 (*ATXN2*), NM_000333 (*ATXN7*), NM_004993 (*ATXN3*), NM_000044 (*AR*) and NM_002111 (*HTT*). The accession numbers of the protein sequences employed were; NP_001007027 (ATN1), NP_000323.2 (ATXN1), NP_002964.2 (ATXN2), NP_000324 (ATXN7), NP_004984 (ATXN3), NP_000035 (AR) and NP_002102 (HTT). Repeat length versus age-at-onset data for each locus was previously collated from published studies [2]. Protein scales were obtained from the ExPASy proteomics server (url: http://www.expasy.ch/). All GC content analyses were performed with custom written software implemented in the Perl programming language. STRIDE, DSSP, and STR secondary structure predictions were performed via the SAM server (url: http://www.soe.ucsc.edu/research/compbio/sam.html). SPSS (version 13) and GraphPad Prism® (version 5) were used for statistical analyses.

## Supporting Information

Figure S1 **Detailed illustration of exponential decay model of the relationship between age at onset and repeat number.** 95% confidence bands (red lines) of regression line describing the relationship between age-at-onset and repeat number for each disorder. Confidence limits of regression line were determined using GraphPad Prism® (version 5).
(TIF)

Figure S2 **The correlation between expandability and toxicity is maintained when using a linear function to describe the relationship between age-at-onset and repeat length.** (**A**) The graph shows the linear regression lines fitted to the age-at-onset and inherited repeat length distributions in the seven polyQ disorders. The inter-locus polyQ toxicities were derived from the parameters of the regression line of each disorder for the modal age-at-onset of 32 years (dashed lines). (**B**) Plot of ranked expandability and ranked inter-locus polyQ toxicity at the modal age-at-onset (32 years) with the regression line. (one-tailed Spearman's rank; *rho* = 0.75; *P* = 0.03; N = 7).
(TIF)

Figure S3 **Correlation of flanking primary amino acid sequence properties with inter-locus polyQ toxicity.** Using a window size of 21 amino acids and a step size of one, locus toxicity was correlated (Spearman's rank) with various physiochemical and compositional characteristics of the primary protein sequence at every amino-acid position flanking the polyQ repeat. Repeat size was normalised to 21 glutamines. The dashed lines represent the threshold for statistical significance (*P*<0.05). As the 3′ sequence of ATXN3 extends just 83 amino acids away from the repeat, all correlations beyond this point involve the remaining six sequences with a correspondingly higher 5% significance threshold. Amino-acid properties were derived from the sources indicated in Table S3. Similar profiles were obtained using sliding window sizes of 15 and 11 amino-acids (data not shown).
(TIF)

Figure S4 **Correlation of predicted flanking secondary protein structure with inter-locus polyQ toxicity.** Using a window size of four amino-acids and a step size of one, inter-locus polyQ toxicity was correlated (Spearman's rank) with the predicted secondary structure as determined from scales of secondary structure formation potential at every amino acid position flanking the polyQ repeat. Repeat size was normalised to 21 glutamines. The dashed lines represent the threshold for statistical significance (*P*<0.05). As the 3′ sequence of ATXN3 extends just 83 amino acids away from the repeat, all correlations beyond this point involve the remaining six sequences with a correspondingly higher 5% significance threshold. Amino-acid properties were derived from the sources indicated in Table S3. Similar profiles were obtained using sliding window sizes of 15 and 11 amino-acids (data not shown).
(TIF)

Figure S5 **Inter-locus polyQ toxicity does not correlate with 1st and 2nd base GC content.** Inter-locus polyQ toxicity does not correlate with 1st and 2nd base GC content, but does correlate with the 3rd base GC content. GC content was sampled using a sliding window of 30 bp (10 codons) and a step size of 3 bp (1 codon). The threshold for statistical significance (dashed lines) is also shown. Only sequences 3′ of the CAG repeat tract were analysed as insufficient sequence is present 5′ of the repeat due to proximity of the repeat tract to the transcription start site at many loci (e.g. *HTT*, *ATXN2*).
(TIF)

Figure S6 **Inclusion of unstable SCA17 alleles strengthens correlation between inter-locus toxicity and expandability.** (**A**) The graph shows the exponential decay regression lines fitted to the age-at-onset and inherited repeat length distributions in the eight polyQ disorders including SCA17. The inter-locus polyQ toxicities were derived from the parameters of the regression line of each disorder for the modal age-at-onset of 30.5 years (dashed lines). (**B**) Plot of ranked expandability and ranked inter-locus polyQ toxicity at the modal age-at-onset (30.5 years) with the regression line (one-tailed Spearman's rank; *rho* = 0.74; *P* = 0.02; N = 8). (**C**) The graph shows the regression analysis between inter-locus polyQ toxicity and the GC content of the genomic DNA flanking sequences at a distance of 100 bp (*r* = −0.77; *P* = 0.01; N = 8).
(TIF)

Table S1 **A. Age-at-death and somatic expandability in MJD patients. B. Age-at-death and somatic expandability in SCA1 patients. C. Levels of somatic expandability are greater in SCA1 than MJD.**
(DOC)

Table S2 A. Somatic expandability in buccal cells of HD patients^1^. B. Somatic expandability^*^ in buccal cells of SCA7 patients^1^. C. Levels of somatic expandability are greater in SCA7 than HD.
(DOC)

Table S3 **Inter-locus polyQ toxicity and expandability of the dynamic DNA polyQ loci.**
(DOC)

Table S4 **Age-at-onset and expandability of unstable SCA17 alleles.**
(DOC)
