# Supplementary figures and images for: Correlation of Inter-Locus Polyglutamine Toxicity with CAG•CTG Triplet Repeat Expandability and Flanking Genomic DNA GC Content
Source: PLoS One. 2011 Dec 6;6(12):e28260. doi: 10.1371/journal.pone.0028260 (PMC3232215; doi:10.1371/journal.pone.0028260)

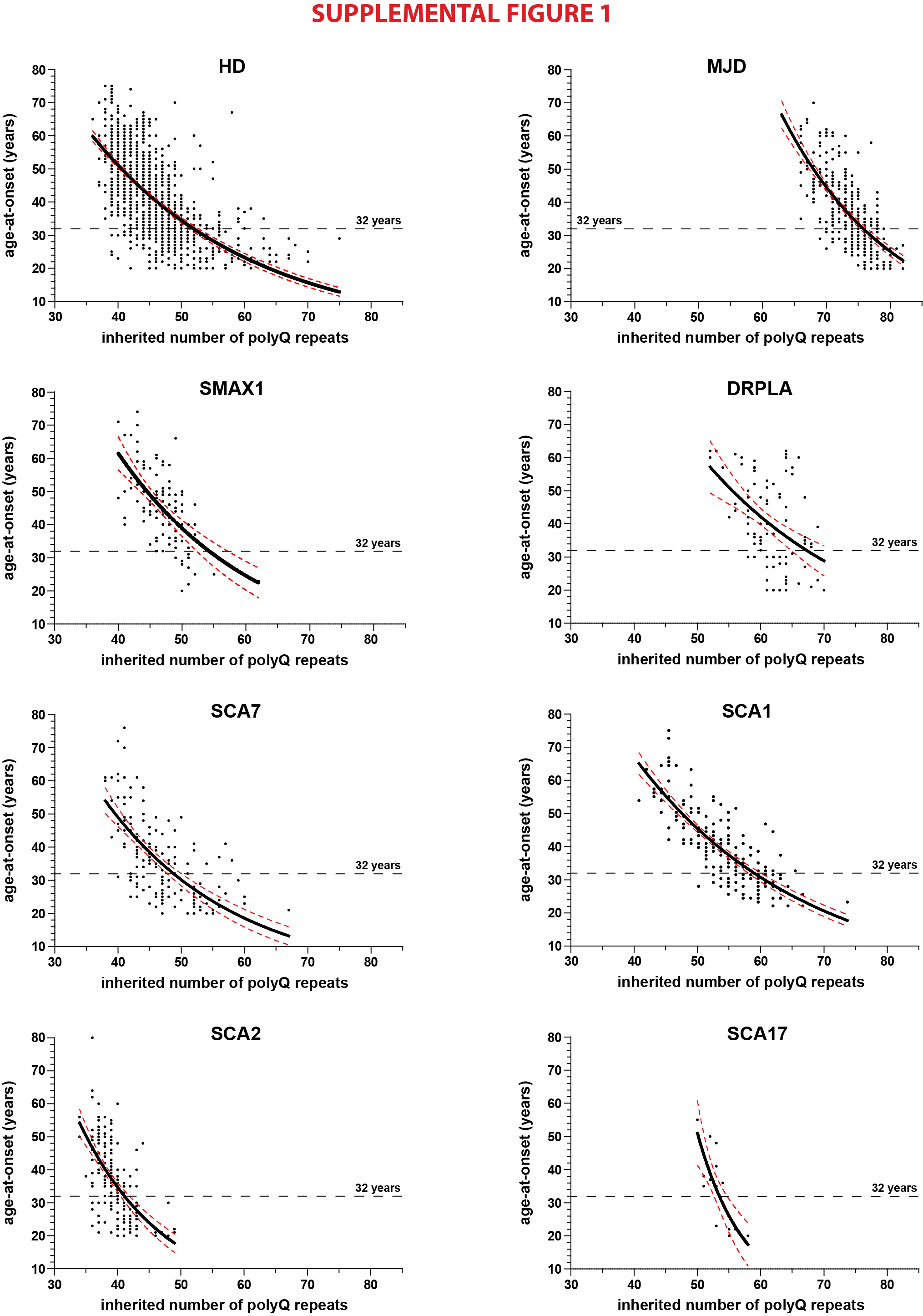

Supplement: Figure S1 — Detailed illustration of exponential decay model of the relationship between age at onset and repeat number. 95% confidence bands (red lines) of regression line describing the relationship between age-at-onset and repeat number for each disorder. Confidence limits of regression line were determined using GraphPad Prism® (version 5). (TIF) [file pone.0028260.s001.tif]

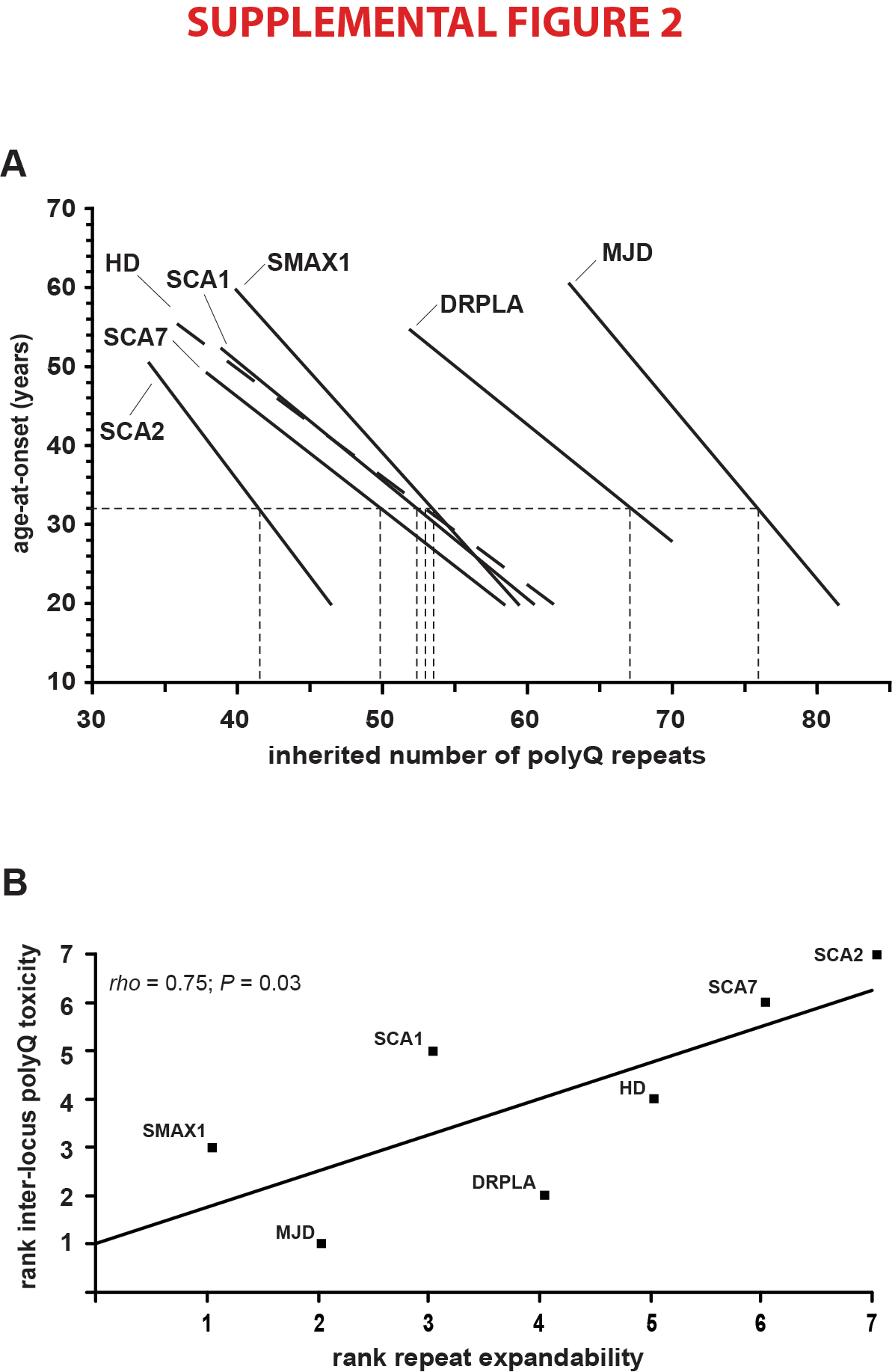

Supplement: Figure S2 — The correlation between expandability and toxicity is maintained when using a linear function to describe the relationship between age-at-onset and repeat length. (A) The graph shows the linear regression lines fitted to the age-at-onset and inherited repeat length distributions in the seven polyQ disorders. The inter-locus polyQ toxicities were derived from the parameters of the regression line of each disorder for the modal age-at-onset of 32 years (dashed lines). (B) Plot of ranked expandability and ranked inter-locus polyQ toxicity at the modal age-at-onset (32 years) with the regression line. (one-tailed Spearman's rank; rho = 0.75; P = 0.03; N = 7). (TIF) [file pone.0028260.s002.tif]

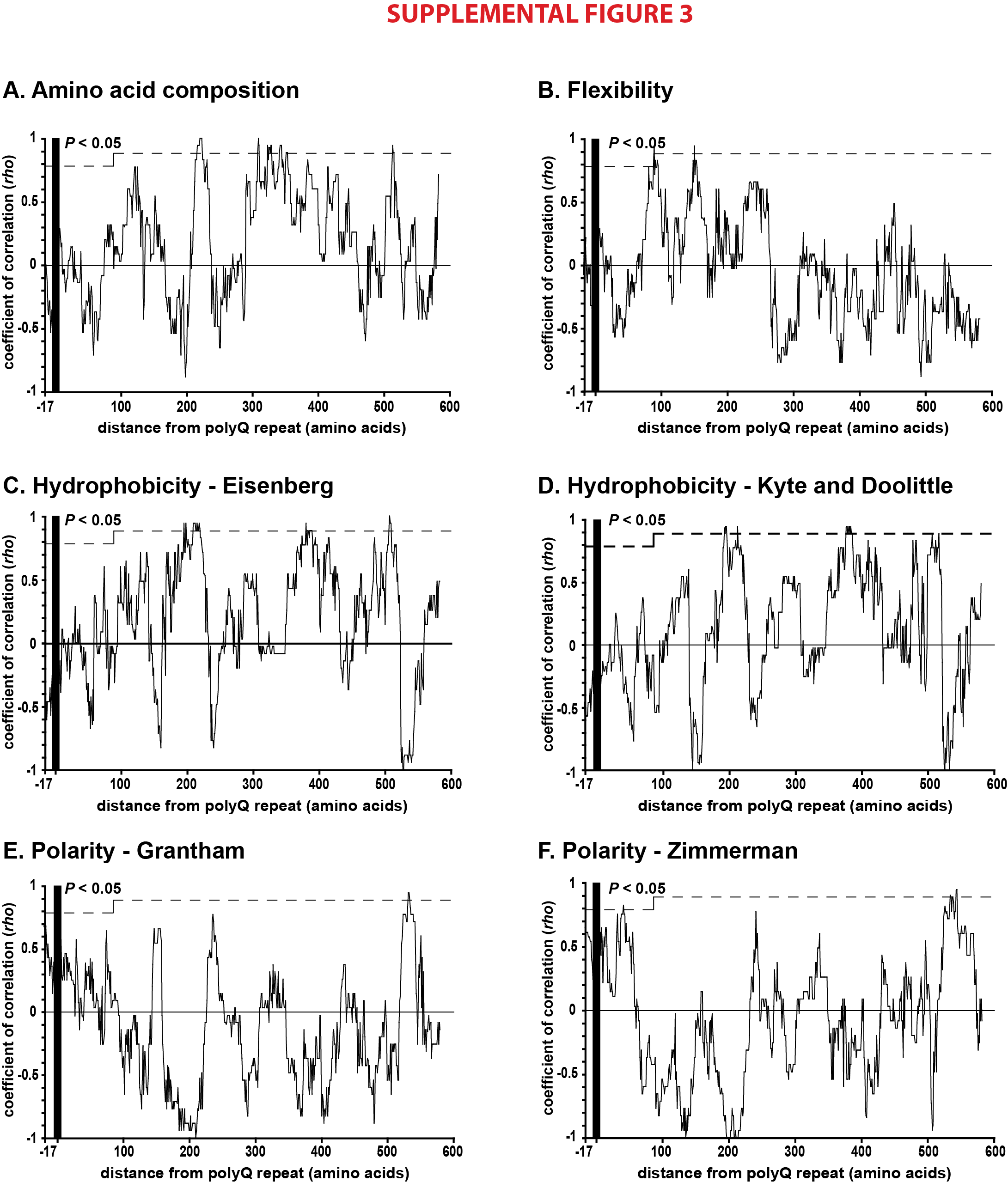

Supplement: Figure S3 — Correlation of flanking primary amino acid sequence properties with inter-locus polyQ toxicity. Using a window size of 21 amino acids and a step size of one, locus toxicity was correlated (Spearman's rank) with various physiochemical and compositional characteristics of the primary protein sequence at every amino-acid position flanking the polyQ repeat. Repeat size was normalised to 21 glutamines. The dashed lines represent the threshold for statistical significance (P<0.05). As the 3′ sequence of ATXN3 extends just 83 amino acids away from the repeat, all correlations beyond this point involve the remaining six sequences with a correspondingly higher 5% significance threshold. Amino-acid properties were derived from the sources indicated in Table S3. Similar profiles were obtained using sliding window sizes of 15 and 11 amino-acids (data not shown). (TIF) [file pone.0028260.s003.tif]

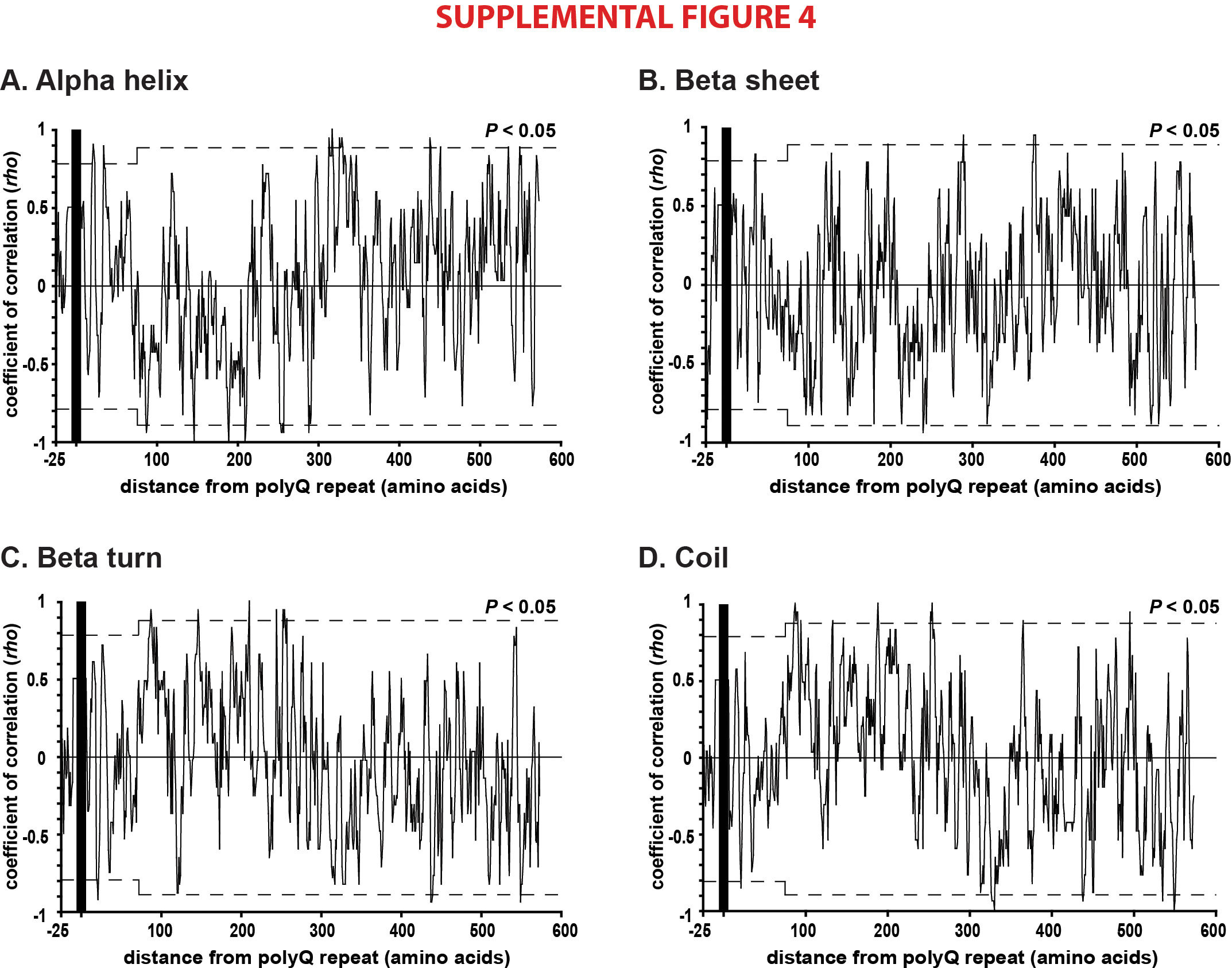

Supplement: Figure S4 — Correlation of predicted flanking secondary protein structure with inter-locus polyQ toxicity. Using a window size of four amino-acids and a step size of one, inter-locus polyQ toxicity was correlated (Spearman's rank) with the predicted secondary structure as determined from scales of secondary structure formation potential at every amino acid position flanking the polyQ repeat. Repeat size was normalised to 21 glutamines. The dashed lines represent the threshold for statistical significance (P<0.05). As the 3′ sequence of ATXN3 extends just 83 amino acids away from the repeat, all correlations beyond this point involve the remaining six sequences with a correspondingly higher 5% significance threshold. Amino-acid properties were derived from the sources indicated in Table S3. Similar profiles were obtained using sliding window sizes of 15 and 11 amino-acids (data not shown). (TIF) [file pone.0028260.s004.tif]

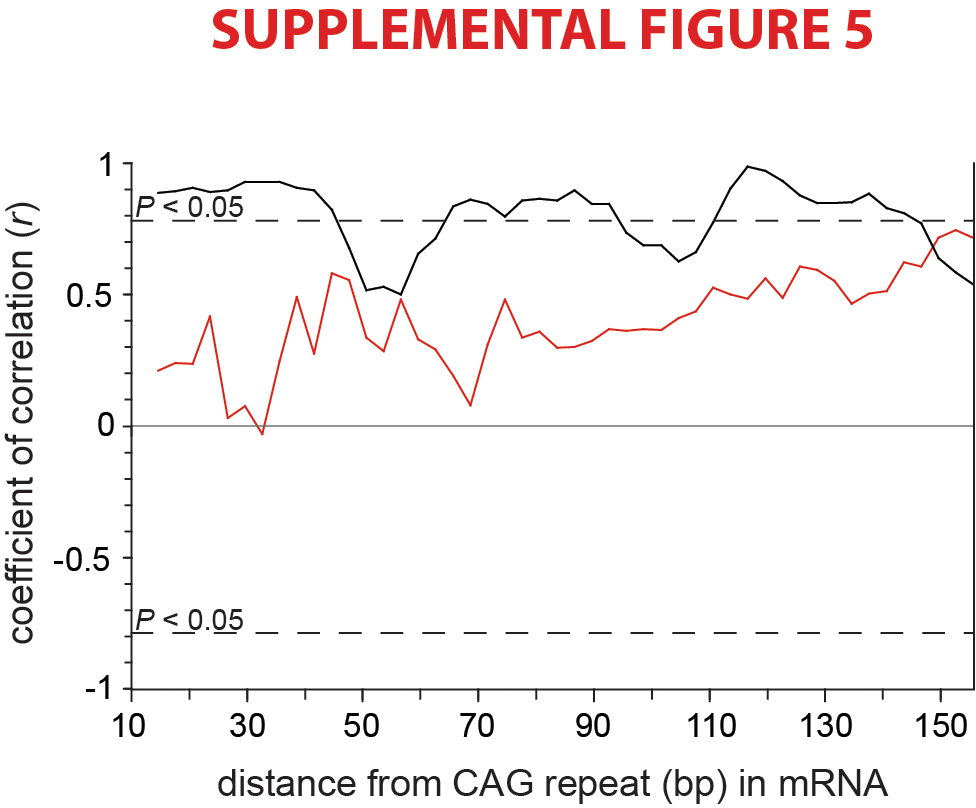

Supplement: Figure S5 — Inter-locus polyQ toxicity does not correlate with 1st and 2nd base GC content. Inter-locus polyQ toxicity does not correlate with 1st and 2nd base GC content, but does correlate with the 3rd base GC content. GC content was sampled using a sliding window of 30 bp (10 codons) and a step size of 3 bp (1 codon). The threshold for statistical significance (dashed lines) is also shown. Only sequences 3′ of the CAG repeat tract were analysed as insufficient sequence is present 5′ of the repeat due to proximity of the repeat tract to the transcription start site at many loci (e.g. HTT, ATXN2). (TIF) [file pone.0028260.s005.tif]

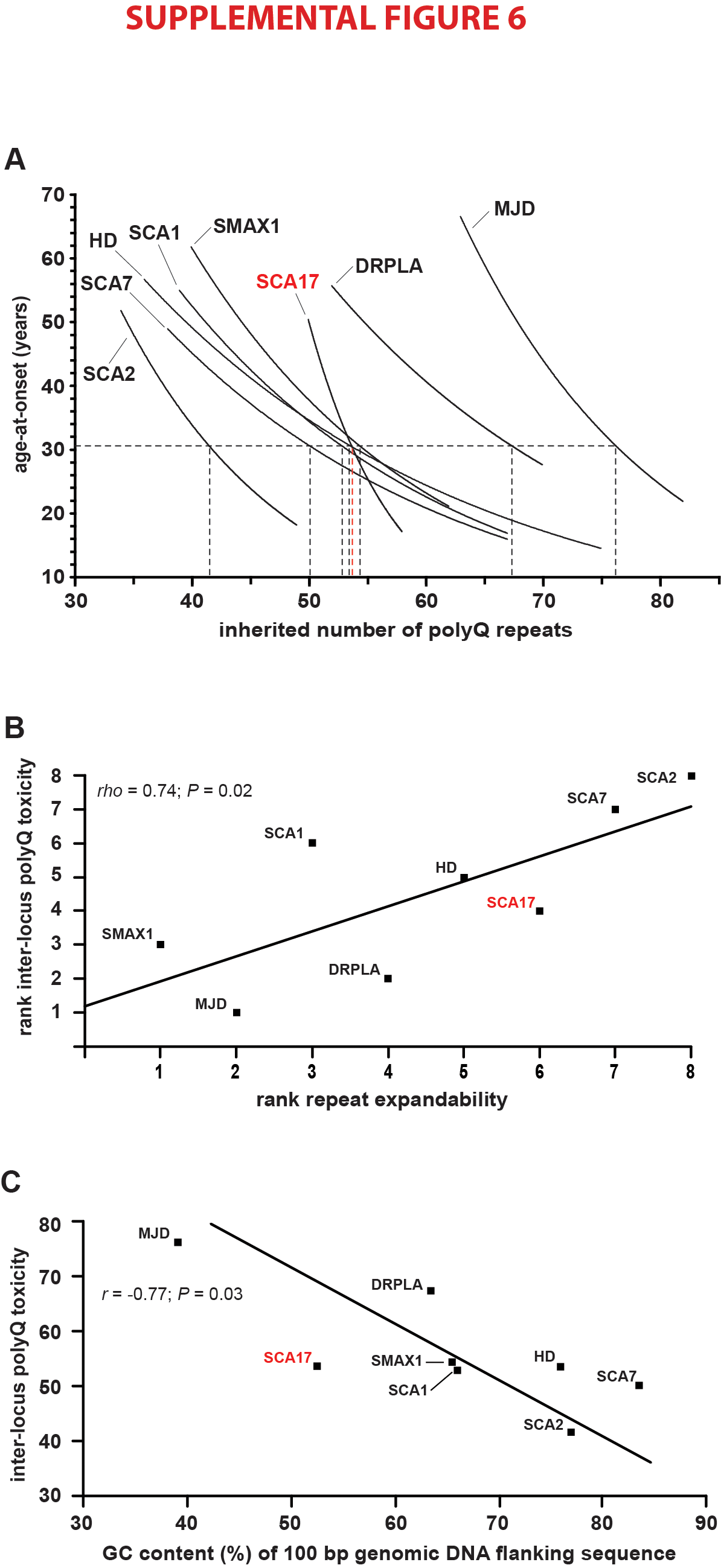

Supplement: Figure S6 — Inclusion of unstable SCA17 alleles strengthens correlation between inter-locus toxicity and expandability. (A) The graph shows the exponential decay regression lines fitted to the age-at-onset and inherited repeat length distributions in the eight polyQ disorders including SCA17. The inter-locus polyQ toxicities were derived from the parameters of the regression line of each disorder for the modal age-at-onset of 30.5 years (dashed lines). (B) Plot of ranked expandability and ranked inter-locus polyQ toxicity at the modal age-at-onset (30.5 years) with the regression line (one-tailed Spearman's rank; rho = 0.74; P = 0.02; N = 8). (C) The graph shows the regression analysis between inter-locus polyQ toxicity and the GC content of the genomic DNA flanking sequences at a distance of 100 bp (r = −0.77; P = 0.01; N = 8). (TIF) [file pone.0028260.s006.tif]
