# Supplementary material for: Correlation of Inter-Locus Polyglutamine Toxicity with CAG•CTG Triplet Repeat Expandability and Flanking Genomic DNA GC Content
Source: PLoS One. 2011 Dec 6;6(12):e28260. doi: 10.1371/journal.pone.0028260 (PMC3232215; doi:10.1371/journal.pone.0028260)
Supplement: Table S1 — A. Age-at-death and somatic expandability in MJD patients. B. Age-at-death and somatic expandability in SCA1 patients. C. Levels of somatic expandability are greater in SCA1 than MJD. (DOC) [file pone.0028260.s007.doc]

**Supplemental Table 1A. Age-at-death and somatic expandability in MJD patients^1^**

| patient | age-at-death | expanded allele^a^ | mean no of bands in cortex^b^ | mean no of bands in white matter^c^ | cortex^d^ (length adjusted) | white matter^e^ (length adjusted) |
| --- | --- | --- | --- | --- | --- | --- |
| 90-274 | 63 | 69 | 10 | 9 | 0.14 | 0.13 |
| 94-547 | 63 | 74 | 10 | 12 | 0.14 | 0.16 |
| M-448 | 62 | 71 | 10 | 10 | 0.14 | 0.14 |
| 93-423 | 58 | 75 | 10 | 11 | 0.13 | 0.15 |
| 94-453 | 56 | 73 | 12 | 10 | 0.16 | 0.14 |
| M-268 | 46 | 75 | 12 | 11 | 0.16 | 0.15 |
| M-318 | 44 | 77 | 10 | 10 | 0.13 | 0.13 |
| M-420 | 43 | 78 | 10 | 10 | 0.13 | 0.13 |
| 88-196 | 27 | 80 | 9 | 10 | 0.11 | 0.13 |
| 2303 | 56 | 75 | 10 |  | 0.13 |  |
| 1965 | 52 | 74 | 9 |  | 0.12 |  |

^1^ data from Maciel et al. 1997 [42].

^b^ average of three experiments

^c^ average of three experiments

^d^ length adjusted somatic expandability (b/a)

^e^ length adjusted somatic expandability (c/a)

**Supplemental Table 1B. Age-at-death and somatic expandability in SCA1 patients^1^**

| patient | age-at-death | expanded allele^a^ | mean no of bands in cortex^b^ | mean no of bands in white matter^c^ | cortex^d^ (length adjusted) | white matter^e^ (length adjusted) |
| --- | --- | --- | --- | --- | --- | --- |
| 94-538 | 77 | 44 | 11 | … | 0.25 |  |
| M-652 | 68 | 45 | 11 | 13 | 0.24 | 0.29 |
| M-378 | 65 | 45 | 11 | 12 | 0.24 | 0.27 |
| 91-288 | 57 | 54 | 15 | 15 | 0.28 | 0.28 |
| 93-441 | 53 | 54 | 16 | 15 | 0.30 | 0.28 |
| 90-276 | 45 | 56 | 11 | 14 | 0.20 | 0.25 |
| 92-271 | 38 | 56 | 13 | 14 | 0.23 | 0.25 |

^1^ data from Maciel et al. 1997.

^b^ average of three experiments

^c^ average of three experiments

^d^ length adjusted somatic expandability (b/a)

^e^ length adjusted somatic expandability (c/a)

**Supplemental Table 1C. Levels of somatic expandability are greater in SCA1 than MJD**

| Tissue | MJD  sample size | SCA1  sample size | U^a^ | *P*-value |
| --- | --- | --- | --- | --- |
| Cortex | 11 | 7 | 0 | 6.3 x 10^-5^ |
| white matter | 9 | 6 | 0 | 0.0004 |

^a^ length adjusted levels of somatic expandability in SCA1 and MJD patients (Tables 2A and 2B) were compared using a Mann-Whitney U-Test
