# Supplementary material for: Correlation of Inter-Locus Polyglutamine Toxicity with CAG•CTG Triplet Repeat Expandability and Flanking Genomic DNA GC Content
Source: PLoS One. 2011 Dec 6;6(12):e28260. doi: 10.1371/journal.pone.0028260 (PMC3232215; doi:10.1371/journal.pone.0028260)
Supplement: Table S2 — A. Somatic expandability in buccal cells of HD patients1. B. Somatic expandability* in buccal cells of SCA7 patients1. C. Levels of somatic expandability are greater in SCA7 than HD. (DOC) [file pone.0028260.s008.doc]

**Supplemental Table 2A. Somatic expandability in buccal cells of HD patients^1^**

| patient | mutation frequency (%) ^a^ | expanded allele ^b^ | buccal cells^c^ |
| --- | --- | --- | --- |
| 2108 | 23.3 | 39 | 0.597436 |
| 13811 | 17 | 39 | 0.435897 |
| 856 | 51.6 | 48 | 1.075 |
| 1757 | 54 | 47 | 1.148936 |
| 1208 | 42.9 | 45 | 0.953333 |
| 1217 | 41 | 44 | 0.931818 |
| 343 | 46.9 | 44 | 1.065909 |
| 1049 | 53.3 | 43 | 1.239535 |
| 7904 | 36.1 | 42 | 0.859524 |
| 576 | 37.7 | 42 | 0.897619 |
| 15679 | 24 | 42 | 0.571429 |
| 2269 | 33.3 | 40 | 0.8325 |

^1^ data from Veitch *et al*. 2007 [8].

^c^ length adjusted somatic mosaicism (a/b)

**Supplemental Table 2B. Somatic expandability* in buccal cells of SCA7 patients1**

| patient | mutation frequency (%)^a^ | expanded allele^b^ | buccal cells^c^ |
| --- | --- | --- | --- |
| ESCA II.5 | 75 | 48 | 1.5625 |

^1^ data from Monckton *et al*. 1999 [34].

^c^ length adjusted somatic mosaicism (a/b)

^*^ Allele repeat number was determined by sizing bands in autoradiograph of a small-pool PCR presented in Monckton *et al*. 1999. band sizing was carried out using SequentiX GelQuest (v2.7.6) gel fragment analysis software. Mutation frequency was calculated as; (total number of mutated alleles/all alleles).

**Supplemental Table 2C. Levels of somatic expandability** are greater in SCA7 than HD

| Tissue | HD  sample size | SCA7  sample size | T^a^ | *P*-value |
| --- | --- | --- | --- | --- |
| Buccal cells | 12 | 1 | -9.578 | > 0.0001 |

^a^ length adjusted levels of somatic mosaicism in HD and SCA7 patients (Tables S3A and S3B) were compared using a one-sample t-test
