# Supplementary material for: Correlation of Inter-Locus Polyglutamine Toxicity with CAG•CTG Triplet Repeat Expandability and Flanking Genomic DNA GC Content
Source: PLoS One. 2011 Dec 6;6(12):e28260. doi: 10.1371/journal.pone.0028260 (PMC3232215; doi:10.1371/journal.pone.0028260)
Supplement: Table S3 — Inter-locus polyQ toxicity and expandability of the dynamic DNA polyQ loci. (DOC) [file pone.0028260.s009.doc]

**Table S3. Inter-locus polyQ toxicity and expandability of the dynamic DNA polyQ loci**

| **Amino Acid Scale** | **Source** |
| --- | --- |
| Amino Acid Composition | http://expasy.org/txt/old-rel/relnotes.51.htm#statistics |
| Hydrophobicity | (Eisenberg *et al.*, 1984) [1] |
| Hydrophobicity | (Kyte and Doolittle, 1982)[2] |
| Polarity | (Grantham, 1974)[3] |
| Polarity | (Zimmerman *et al.*, 1968)[4] |
| Alpha-helix | (Chou and Fasman, 1978)[5] |
| Beta-turn | (Chou and Fasman, 1978)[5] |
| Beta-sheet | (Chou and Fasman, 1978)[5] |
| Average flexibility | (Bhaskaran and Ponnuswamy, 1984)[6] |
| Coil | (Deleage and Roux, 1987)[7] |

**References**

1. Eisenberg D, Schwarz E, Komaromy M, Wall R (1984) Analysis of membrane and surface protein sequences with the hydrophobic moment plot. J Mol Biol 179: 125-142.

2. Kyte J, Doolittle RF (1982) A simple method for displaying the hydropathic character of a protein. J Mol Biol 157: 105-132.

3. Grantham R (1974) Amino acid difference formula to help explain protein evolution. Science 185: 862-864.

4. Zimmerman JM, Eliezer N, Simha R (1968) The characterization of amino acid sequences in proteins by statistical methods. J Theor Biol 21: 170-201.

5. Chou PY, Fasman GD (1978) Prediction of the secondary structure of proteins from their amino acid sequence. Adv Enzymol Relat Areas Mol Biol 47: 45-148.

6. Bhaskaran R, Ponnuswamy PK (1984) Dynamics of amino acid residues in globular proteins. Int J Pept Protein Res 24: 180-191.

7. Deleage G, Roux B (1987) An algorithm for protein secondary structure prediction based on class prediction. Protein Eng 1: 289-294.
