# Supplementary material for: Correlation of Inter-Locus Polyglutamine Toxicity with CAG•CTG Triplet Repeat Expandability and Flanking Genomic DNA GC Content
Source: PLoS One. 2011 Dec 6;6(12):e28260. doi: 10.1371/journal.pone.0028260 (PMC3232215; doi:10.1371/journal.pone.0028260)
Supplement: Table S4 — Age-at-onset and expandability of unstable SCA17 alleles. (DOC) [file pone.0028260.s010.doc]

| Source | Repeat  length | Age-at-onset | Generation | Sex | Transmission | Expandability^a, c^ |
| --- | --- | --- | --- | --- | --- | --- |
| Rassmussen (2007) | 54 | 36 | F1 | M |  |  |
|  | 55 | 20 | F2 | F | 1 | 0.05^b^ |
|  | 61 | 11 | F2 | F | 2 | 0.37^b^ |
|  | 53 | 48 | F1 | M |  |  |
|  | 58 | 20 | F2 | M | 3 | 0.28^b^ |
|  | 56 | 22 | F2 | M |  |  |
|  | 51 | 38 | F1 | F |  |  |
|  | 51 | 35 | F2 | M | 4 | 0.00 |
|  | 52 | 37 | F2 | F | 5 | 0.06 |
|  | 55 | 16 | F2 | F |  |  |
| Zuhlke (2001) | 53 | 34 | F1 | F |  |  |
|  | 55 | 20 | F2 | F | 6 | 0.11 |
| Zuhlke (2005) | 49 |  | F1 | M |  |  |
|  | 52 | 50 | F2 | F | 7 | 0.21^b^ |
|  | 53 | 41 | F2 | M | 8 | 0.29^b^ |
| Maltecca (2001) | 53 | 35 | - | F |  |  |
|  | 53 | 34 | F1 | M |  |  |
|  | 53 | 23 | - | M |  |  |
|  | 66 | 3 | F2 | F | 9 | 0.72^b^ |
| Gao (2008) | 50 | 55 | - | F |  |  |
|  | 55 | 22 | - | F |  |  |

**Table S4. Age-at-onset and expandability of unstable SCA17 alleles**

^a^ Expandability calculated as per Brock *et al* (1999) [21]; expandability = intergenerational length change/(progenitor allele length- 35)

^b^ Male transmissions.

^c^ Median expandability = 0.17
